# Effect of Natural Ionizing Radiation on Health Indicators in Region with Monazite Sand in Brazil

**DOI:** 10.1055/s-0044-1791695

**Published:** 2024-10-25

**Authors:** José Ulisses Manzzini Calegaro, Luiz Augusto Casulari, Marcos Tadeu D'Azeredo Orlando

**Affiliations:** 1 Research and Innovation Department, Hospital de Base, Brasília, DF, Brazil; 2 Health Sciences Post-Graduation, Universidade de Brasília, Brasília, DF, Brazil; 3 Physics Department, Universidade Federal do Espírito Santo, Vitória, ES, Brazil

**Keywords:** cancer, germinal damage, health indicators, monazite sand, natural ionizing radiation

## Abstract

**Objective** This article compares the occurrence of cancer and germinal damage in the city of Guarapari-Espírito Santo (ES), an area with high natural ionizing radiation, with other coastal cities.

**Material and Methods** The evaluated cities were: Guarapari (ES), Campos (Rio de Janeiro), Rio Grande (Rio Grande do Sul), and Ilhéus (Bahia); the following factors were considered: mortality rate % from tumors (2007–2017), mortality rate % from tumors up to 15 years of age (2007–2017), hospital morbidity rate % from tumors up to 15 years of age (2008–2018), and hospital morbidity rate % from congenital malformation and chromosomal anomalies (2008–2018). Radiometric surveys were conducted by the Applied Physics Group at Universidade Federal do Espírito Santo.

**Results** The natural radiation levels recorded in Guarapari resulted in accumulated dose between 3.65 and 10.95 mSv/year, and 1 mSv in the other cities. The highest average cancer mortality rates were: Rio Grande = 22.4%; Guarapari = 17.6%; Campos = 16.7%; and Ilhéus = 11.8%. The proportional hospital mortality and morbidity rates for cancer up to 15 years of age are as follows: Ilhéus = 3.36 and 5.87%; Rio Grande = 0.79 and 7.38%; Guarapari = 0.64 and 7.25%; and Campos = 0.39 and 9.13%. The hospital morbidity rate due to congenital malformations and chromosomal anomalies was 0.72% for Campos, 0.63% for Rio Grande, 0.62% for Guarapari, and 0.43% for Ilhéus.

**Conclusion** There was no increase in cases of cancer or germ damage in Guarapari. These results indicated a dose threshold in the induction of these damages, contradicting the current linear no-threshold theory.

## Introduction

Radiation has been used over a century for diagnostic purposes, primarily through radiodiagnostics, and therapeutically as in radiotherapy. After the occurrence of harmful events related to radiation, there was a need to define exposure limits to ionizing radiation so that occupation activities related to it could be safely conducted. As the available information was not precise, the effects of high or moderate doses absorbed by biological systems were extrapolated to point zero, assuming a linear (or equivalent) relationship between dose and response. Considering this assumption, any radiation dose above zero can result in a biological effect, constituting a potential health risk. This theory (linear no-threshold [LNT]), so-called conservation, is still in force. 1

The original work by Muller in 1927, with X-ray irradiation of *Drosophila melanogaster* (fruit fly) showing genetic damage, was the starting point for regulatory agencies—from 1930 onwards—to establish the theory of linear extrapolation of high and medium ionizing radiation levels in biological systems to point zero, without threshold. 2 3 This was one of the supports for the abovementioned theory, with greater emphasis on the induction of cancer through exposure to low ionizing radiation levels .

The biological effects of ionizing radiation have been classified in two types: nonstochastic or of more immediate nature and stochastic or cumulative in nature. Furthermore, they can be categorized as genetic, of somatic nature (that is, they affect the human body) or of germinal nature (that is, they can be transmitted). The greatest impact on human beings was the nuclear explosions in Hiroshima and Nagasaki, at the end of the Second World War, when thousands of people were killed by the shock produced, or heat generated, or high levels of radiation emitted by the fission of the radioactive material.

The assessment of biological indicators in areas with high natural ionizing radiation levels has become a valuable element in this issue because it moves from the theoretical realm to an analysis of evidence-based medicine. 4 Radiometric surveys were initially reported by Roser and Cullen for the territory of monazite sands on the coast of the state of Espírito Santo (ES), more specifically in Guarapari and its district Meaípe, 5 which revealed the highest natural radiation levels due to the thorium-232 cycle in Brazil.

The objective of this study was to analyze the hypothesis that cumulative exposure to radiation in the monazite sands of the municipality of Guarapari could lead an increase in health indicators related to cancer or congenital malformation.

## Methods

This study has analytical cross-sectional design with data collected between 2007 and 2017. Data collection sites were DATASUS (an agency of the Unified Health System) and IBGE (Brazilian Institute of Geography and Statistics), both federal government agencies. The evaluated cities were: Guarapari (ES), Campos (Rio de Janeiro [RJ]), Rio Grande (Rio Grande do Sul), Ilhéus (Bahia), located on the Brazilian coast, with similar socioeconomic profiles (Human Development Index [HDI]), equivalent population pyramids, and located at sea level.

The analyzed variables included the mortality percentage from tumors (2007–2017), mortality percentage up to 15 years of age (2007–2017), hospital morbidity percentage up to 15 years of age (2008–2018), and hospital morbidity percentage due to congenital malformations and chromosomal anomalies (2008–2018). Data were extracted from the health information booklet on municipalities annually published by DATASUS, with percentage numbers, and are organized with the collaboration of the technical staff from the Ministry of Health library.

The natural radiation assessment methodology followed data from the Applied Physics Group of the Federal University of Espírito Santo 6 because considerable changes had occurred in many locations surveyed by Roser and Cullen 5 : dirt roads were paved, new roads were built, and cement floors were installed inside the houses. In the work of this group, sand samples were dried in an oven at approximately 100°C for 24 hours, sieved, and stored in sealed plastic containers. The Gamma-Scout gamma ray detector was calibrated on a scale from 0.01 to 1000.00 µSv, with conversion of the dose rate based on Cs-137. The sand composition of beaches of Guarapari-ES-Brazil was analyzed by X-ray diffraction, indicating the presence of thorium and uranium as radioactive elements. Thorium-223, the main component of these sands with half-life = 11.4 × 10 ^10^ years, undergoes successive decays for unstable elements, finally resulting in lead-208 (the final stable element). From the others locations, Campos, Ilhéus, and Rio Grande, the considered level was the permissible environmental background radiation for the general population. 7 The analysis' primary outcome was the number of neoplasms and congenital malformation among analyzed cities.

Nonparametric mean tests were conducted using the Mann–Whitney method after checking for normality with the Kolmogorov test. Values were considered significant for *p* ≤ 0.05.

## Results

The natural radiation levels recorded on the beaches of Guarapari indicate doses ranging from 10 to 30 μSv/h. Considering exposure of 1 hour per day for 365 days, this results in annual accumulated dose ranging from 3.95 to 10.95 mSv/year. Considering the average population lifespan of 70 years, the individual cumulative radiation varies from 255.5 mSv to 3.99 Sv. 6

The considered level of natural radiation for Campos, Ilhéus, and Rio Grande was 1 mSv/year. 7

Table 1 shows the population, HDI, and neonatal mortality per thousand live births in the four cities. It was observed that the population of Campos is much larger than that of the other cities. On the other hand, the population of Guarapari is the smallest. HDI is lower in Ilhéus compared with the other cities, which have similar HDI. Regarding neonatal mortality, Ilhéus has the highest rate and Guarapari the lowest.

**Table 1:** Information about the compared cities – census of 2010

	Ilhéus (BA)	Guarapari (ES)	Campos (RJ)	Rio Grande (RS)
Population	162.334	105.286	463.731	197.228
HDI	0.690	0.731	0.716	0.744
Neonatal mortality	17.97	10.72	13.74	11.44

Abbreviations: BA, Bahia; ES, Espírito Santo; HDI, Human Development Index; RJ, Rio de Janeiro; RS, Rio Grande do Sul.

In Table 2 , the mortality rate from tumors in the four cities is presented. Over the 10 years of analysis, the average mortality rate is higher in the city of Rio Grande, and the lowest in Ilhéus. Guarapari and Campos have similar mortality rates. Furthermore, the mortality rate did not change during the 10 years of observation in each city.

**Table 2:** % mortality due to tumors

	2007	2008	2009	2010	2011	2012	2013	2014	2015	2016	2017	Average
Campos (RJ)	15.9	17.7	15.6	16.5	16.3	16.3	17.2	17.4	16.8	16.7	17.35	16.72
Rio Grande (RS)	21.2	25.4	21.9	23.2	21.8	22.7	24.1	22.7	21.6	21.0	21.1	22.42
Ilhéus (BA)	12.9	12.4	12.8	12.1	9.6	11.7	9.5	12.4	11.3	11.7	13.4	11.8
Guarapari (ES)	16.0	16.1	17.1	14.3	17.0	18.7	20.2	18.2	18.6	18.7	20.3	17.6

Abbreviations: BA, Bahia; ES, Espírito Santo; RJ, Rio de Janeiro; RS, Rio Grande do Sul.

Table 3 shows the mortality rate from tumors in childhood and adolescence up to 15 years of age in the four cities. The lowest mortality rate was found in Campos and the highest in Ilhéus. Guarapari and Rio Grande have similar results. In Ilhéus, mortality rates showed high variability during the period, ranging from 0 to 4.4%.

**Table 3:** Mortality % from tumors in childhood and adolescence (up to 15 years)

	2007	2008	2009	2010	2011	2012	2013	2014	2015	2016	2017	Median
Campos (RJ)	0.25	0.25	0.27	0.75	0.33	0.50	0	0.24	0.25	1.21	0.25	0.39%
Rio Grande (RS)	1.24	0.42	1.08	1.01	1.78	0.33	1.03	0.20	0.60	1.1	0.9	0.79%
Ilhéus (BA)	4.4	2.01	1.3	2.15	0	1.44	0	1.8	1.26	0	0.34	3.36%
Guarapari (ES)	0	2.38	0	0	0	0	1.6	1.6	0.8	0.7	1.28	0.64%

Abbreviations: BA, Bahia; ES, Espírito Santo; RJ, Rio de Janeiro; RS, Rio Grande do Sul.

Table 4 shows that morbidity from tumors in childhood and adolescence up to 15 years of age is lower in Ilhéus and higher in Campos. The frequencies in Rio Grande and Guarapari are similar. It was observed that there was a worsening of this parameter between 2013 and 2018 in Campos. On the other hand, in Ilhéus, improvement has been observed in recent years since 2012. In Guarapari and Rio Grande, the percentage remained similar during the observation period.

**Table 4:** Hospital morbidity % from tumors in childhood and adolescence (up to 15 years)

	2008	2009	2010	2011	2012	2013	2014	2015	2016	2017	2018	Median
Campos (RJ)	6.30	6.64	7.17	7.68	7.94	9.39	10.75	10.07	10.88	11.47	12.21	9.13%
Rio Grande (RS)	7.81	8.46	8.05	7.02	7.61	7.60	7.83	6.83	6.67	6.57	6.73	7.38%
Ilhéus (BA)	7.68	6.7	5.31	7.0	5.34	5.26	5.42	5.91	6.21	4.20	5.63	5.87%
Guarapari (ES)	6.42	7.08	6.34	5.7	6.9	8.13	8.28	7.6	7.44	7.62	7.77	7.25%

Abbreviations: BA, Bahia; ES, Espírito Santo; RJ, Rio de Janeiro; RS, Rio Grande do Sul.

Assessments of morbidity percentages due to congenital malformation and chromosomal anomalies are presented in Table 5 . The lowest percentage was found in Ilhéus, similar percentage was found between Guarapari and Rio Grande, and slightly higher percentage was found in Campos. There was no significant change over the years in these cities.

**Table 5:** Hospital morbidity % from congenital malformations and chromosomal anomalies

	2008	2009	2010	2011	2012	2013	2014	2015	2016	2017	2018	Median
Campos (RJ)	0.71	0.44	0.82	0.86	0.68	0.70	0.73	0.60	0.55	0.66	0.45	0.72
Rio Grande (RS)	0.59	0.58	0.64	0.82	0.73	0.74	0.65	0.65	0.62	0.48	0.4	0.63
Ilhéus (BA)	0.40	0.49	0.39	0.52	0.58	0.53	0.34	0.56	0.48	0.26	0.24	0.43
Guarapari (ES)	0.64	0.61	0.63	0.69	0.59	0.53	0.69	0.65	0.63	0.56	0.53	0.62

Abbreviations: BA, Bahia; ES, Espírito Santo; RJ, Rio de Janeiro; RS, Rio Grande do Sul.

None of the evaluated indicators showed statistically significant difference, especially in Guarapari.

## Discussion

The cancer indicators analyzed over 11 years in Guarapari, after careful dosimetry performed for a decade by the Physics Group of Pontifícia Universidade Católica do Rio de Janeiro, updated by the Applied Physics Group of Universidade Federal do Espírito Santo and supported by data from the Unified Health System, did not show increase of the disease compared with the other locations used for comparison. Furthermore, in the population up to 15 years of age, considered more sensitive to the biological effects of ionizing radiation, there is a trend of lower values in records. The cumulative radiation exposure for individuals aged 70 years is between 255.5 and 3990 mSv for Guarapari and Meaípe; however, these assessments do not consider the radioactivity incorporation by food, an aspect emphasized in Brazil since 1970, 8 which would surely increase these values.

It is possible to observe that the lowest neonatal mortality rate among evaluated locations was observed in Guarapari. The correlation between breast cancer and radiation levels in Guarapari, compared with the other cities in the state of ES, was the lowest found between the years 2008 and 2013. 9 The trend of current indicators, as well as of others already disclosed, 10 may reinforce the existing theory of hormesis due to exposure to low ionizing radiation levels .

An interesting evaluation was conducted on workers in the American navy, distributed into three groups and followed for 13 years, in which individuals were submitted to exposure ranging from 5 to 10 mSv/year and were compared with a nonexposed group. The conclusion was that there was no higher frequency of leukemia, hematopoietic tumors, or lung tumors. Surprisingly, the occurrence was even lower compared with the control group—could it be a protective effect of these radiation levels. 11

India, along with Brazil, is another geographical area with monazite sands, especially in the Kerala province. Assessment of cancer incidence was conducted in Karunagappally, a city in the region, with population exposure of 75 mSv/year. The results showed that this occurrence is equivalent to other areas with levels considered normal. 12 A subsequent cohort study in this city for 10.5 years also showed no increase in cancer incidence. 13 It is noteworthy mentioning that the cumulative radiation for each individual with 70-year lifespan is 5,250 mSv.

A cohort study was conducted in Yangjiang, province of Guangdong, China, from 1979 to 1995, with average exposure of 6.4 mSv per inhabitant. The study showed that cancer mortality was slightly lower than that of the control population, considered as protective effect of ionizing radiation. 14 The continuation of these studies, covering the period from 1979 to 1998, that is, 19 years, confirmed this information and demonstrated similar results for noncancer mortality. 15 The cumulative radiation for an individual with average age of 70 years is 448 mSv.

Genetic damages were initially reported by Herman Miller, awarded the Nobel Prize in Medicine in 1946, but contested by more recent publications for having omitted information from his geneticist. 16 17 These effects at cellular level can occur with low ionizing radiation levels and cause damage to deoxyribonucleic acid, either due to direct effects or to the accumulation of epigenetic changes that would later result in tumor induction, despite the existence of repair systems. On the other hand, these damage mechanisms could lead to deleterious germline mutations, although never observed in humans at these levels. 18 These are the premises adopted by agencies such as the United Nations and the Nuclear Regulatory Commission (American regulatory agency). These levels were not observed in the population of Guarapari, even with secular irradiation that accumulated between 1,095 and 3,285 mSv since their foundation.

**Fig. 1:**
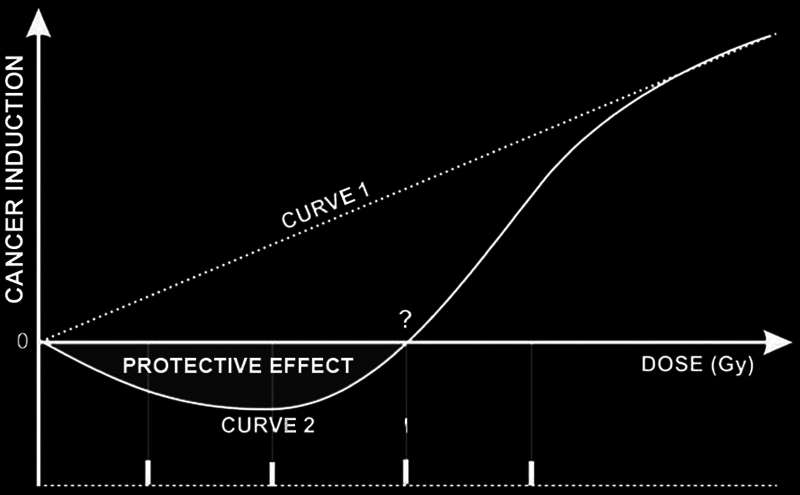
Representative curves of cancer induction. Curve 1 no threshold; curve 2 threshold and probable protective effect.

Data from this work corroborate previous results 19 and safely allow the exclusion of the LNT theory. This theory serves bureaucratic, political, economic, and military convenience and is grounded in widespread public misinformation, as has been recently pointed out. 20 21 22 Current information suggests the existence of a threshold in these potential harmful occurrences, which will be established from cumulative doses as proposed in this study. Fig. 1 shows two curves: 1 - no-threshold linear; 2 - threshold and a negative area under the horizontal axis with regression to point zero, suggesting a protective effect from radiation at these levels. 19 23 The limits for population exposure rates may also be based on similar principles. Measurements in areas with higher natural radiation levels should support most conclusions about their effects on humans, rather than mathematical and statistical extrapolations of cell culture, tissues, and organisms that show questionable biological equivalence with humans.

It is possible to observe that the lowest neonatal mortality rate among evaluated locations occurs in Guarapari. The correlation between breast cancer and radiation levels in Guarapari, compared with the other cities in the state of ES, was the lowest found between the years 2008 and 2013. 22 The trend of current indicators, as well as of others already disclosed, 17 may reinforce the existing theory of hormesis due to exposure to low ionizing radiation levels .

## Conclusion

There was no increase in overall tumor percentage, percentage of tumors up to 15 years of age, and hospital morbidly from tumors up to 15 years of age regarding the occurrence of somatic damage in the population of Guarapari with activities ranging from 3.65 to 10.95 mSv/year. At the germinative level, there was also no higher hospital morbidity from congenital malformations or chromosomal aberrations, even with secular accumulation (considering 300 years of history) between 1,095 and 3,285 mSv of ionizing radiation.

Data suggest that there is a dose threshold or dose rate in cancer induction or germinal damage, formally contradicting the no-threshold linear theory.
